# Correction: FOXO3a-dependent regulation of Puma in response to cytokine/growth factor withdrawal

**DOI:** 10.1084/jem.2006035301132021c

**Published:** 2021-01-19

**Authors:** Han You, Marc Pellegrini, Katsuya Tsuchihara, Kazuo Yamamoto, Georg Hacker, Miriam Erlacher, Andreas Villunger, Tak W. Mak

Vol. 203, No. 7 | 10.1084/jem.20060353 | June 26, 2006

The authors regret that a reference related to Fig. 2 A was missing in the original version of this paper. Data in Fig. 2 A were previously published in a 2006 *PNAS* article. The same data were reported again in the *JEM* article to describe the background of the experimental design. The missing sentence with the You et al. (2006) citation is in bold below.

Figure 2. Puma is a FOXO3a transcriptional target gene. (A) **This experimental design and data were previously published in You et al. (2006).** p53^+/+^ and p53^−/−^ FOXO3a TM-ER MEFs were exposed to 4-OHT (0.5 µM) for 6 h, and lysates were subjected to Western blotting using antibodies directed against the indicated proteins.

You, H., K. Yamamoto, and T.W. Mak. 2006. Regulation of transactivation-independent proapoptotic activity of p53 by FOXO3a. *Proc. Natl. Acad. Sci. USA.* 103:9051–9056.

